# Habitat-Adapted Fungal Symbionts Promote Salt Stress Tolerance Through Distinct Root Mechanisms and Shared Shoot Regulatory Networks in *Arabidopsis thaliana*

**DOI:** 10.3390/ijms27177590

**Published:** 2026-08-25

**Authors:** Silvia Martínez-Fenoll, Adrián González Ortega-Villaizán, Estefanía Rodríguez-Dobreva, Luis Morales-Quintana, Patricio Ramos, Jesús Vicente-Carbajosa, Rosario Haro, Begoña Benito, Stephan Pollmann

**Affiliations:** 1 Center for Plant Biotechnology and Genomics, National Institute of Agricultural Research and Technology and Food (INIA/CSIC), Polytechnic University of Madrid (UPM), Montegancedo Campus, 28223 Madrid, Spain; silvia.martinezfenoll@upm.es (S.M.-F.); adrian.gonzalezort@upm.es (A.G.O.-V.); estefania.rdobreva@upm.es (E.R.-D.); jesus.vicente@upm.es (J.V.-C.); rosario.haro@upm.es (R.H.); begona.benito@upm.es (B.B.); 2 Multidisciplinary Agroindustry Research Laboratory, Instituto de Ciencias Biomédicas, Facultad de Ciencias de la Salud, Universidad Autónoma de Chile, Cinco Poniente #1670, Talca 3460000, Chile; luis.morales@uautonoma.cl; 3 Plant Microorganism Interaction Laboratory, Instituto de Ciencias Biológicas, Universidad de Talca, Talca 3460787, Chile; pramos@utalca.cl; 4 Departamento de Biotecnología-Biología Vegetal, Escuela Técnica Superior de Ingeniería Agronómica, Alimentaria y de Biosistemas, Universidad Politécnica de Madrid (UPM), 28040 Madrid, Spain

**Keywords:** symbiosis, growth promotion, plant–fungus interaction, plant performance, salt stress resilience, holobiont

## Abstract

Salinity is a major constraint to crop productivity. Beneficial plant–fungus interactions represent a promising strategy to enhance stress resilience. Here, we investigated fungal endophytes isolated from the roots of *Oryza sativa* cultivated in saline-prone marshlands of the Guadalquivir River, Spain. From a collection of 38 isolates, five salt-tolerant strains exhibiting plant growth-promoting activity were identified, including a previously uncharacterized *Reticulascus* sp. strain S5. Co-cultivation assays with the non-native host plant *Arabidopsis thaliana* demonstrated that S5 increased the root and shoot biomass under salt stress. To elucidate the underlying molecular mechanisms, a comprehensive RNA-Seq analysis of the roots and shoots under control and saline conditions was performed. Fungal colonization induced pronounced transcriptomic changes, particularly in the shoots, including rewiring of the auxin- and abscisic acid-related pathways and the induction of genes associated with cell wall remodeling. Concurrently, defense-related processes, including glucosinolate biosynthesis and ethylene signaling, were broadly repressed, suggesting attenuated stress perception in colonized plants. In the roots, S5 inoculation suppressed the expression of genes involved in root hair development and cell wall organization, indicating a fungus-driven reconfiguration of root development. Moreover, comparative analysis with *Fusarium* sp. K-23, a fungus that has previously been demonstrated to promote plant growth under salinity stress, revealed distinct root-associated mechanisms but convergence on a shared regulatory module in shoots involving ABA-responsive transcription factors and osmotic stress regulators. Collectively, our findings demonstrate that *Reticulascus* sp. S5 enhances plant salt stress tolerance through the coordinated transcriptional reprogramming of growth, hormone signaling, and stress responses, highlighting a possible potential of habitat-adapted endophytes for sustainable crop improvement.

## 1. Introduction

Soil salinization constitutes a significant environmental challenge that restricts agricultural productivity on a global scale. Current estimates indicate that salinity affects over 20% of irrigated land, a proportion anticipated to rise due to climate change, sea-level rise, and unsustainable irrigation practices [1,2]. Salinity stress imposes osmotic and ionic pressures on plants, resulting in decreased water uptake, ion toxicity, and the disruption of cellular homeostasis. These effects compromise essential physiological functions, such as photosynthesis, nutrient acquisition, and growth, ultimately leading to reduced biomass accumulation and yield [3]. At the cellular level, excessive sodium (Na^+^) and chloride (Cl^−^) ions disrupt enzymatic activities and compromise membrane integrity, while osmotic stress induces stomatal closure, thereby limiting carbon assimilation [4]. Furthermore, salinity triggers the overproduction of reactive oxygen species (ROS), which cause oxidative damage unless effectively neutralized by antioxidant systems [5]. Although plants have evolved complex adaptive mechanisms to deal with salinity stress, the triggered responses are often inadequate or insufficient to cope with prolonged or severe stress conditions [6,7]. Therefore, enhancing plant resilience to salinity remains a critical goal for sustainable agriculture.

In recent years, there has been a growing focus on the role of plant-associated microorganisms in mitigating abiotic stress. Symbiotic plant–microbe relationships are considered supplementary adaptive mechanisms that can lead to sustained enhancement of the stress resistance of the host plants. Fungal endophytes, which are fungi that colonize plant tissues without inducing disease, have been identified as significant contributors to plant fitness under challenging environmental conditions [8]. These beneficial plant–fungus interactions can enhance nutrient acquisition, improve water use efficiency, and modulate plant stress responses, thereby facilitating growth and survival under stress [9,10,11]. For example, *Microsphaeropsis arundinis* was reported to protect salt-sensitive wheat against salt stress [12], and some other dark septate fungal endophytes were reported to mitigate salt stress in cowpea plants [13]. Moreover, the fungal root-colonizing endosymbiont *Serendipita indica* has recently been reported to mitigate salt stress by evacuating salt from the root [14,15]. The concept of habitat-adapted symbiosis has gained prominence, suggesting that plants in extreme environments host microbial partners that impart stress tolerance traits [16]. Studies have shown that fungal endophytes isolated from plants inhabiting saline, arid, or otherwise challenging habitats can transfer stress tolerance to non-native host plants, suggesting a conserved and transferable mechanism of action [17]. These findings highlight the importance of the plant holobiont, which describes the integrated system of plant and associated microbiota, in shaping plant performance under environmental stress [18].

The advantageous effects of fungal endophytes are frequently facilitated through the significant reprogramming of plant physiological and molecular processes, particularly those associated with hormone metabolism and signaling. Plant hormones, such as auxins, abscisic acid (ABA), ethylene, and jasmonates, are pivotal in regulating growth, development, and stress responses [19]. Fungal symbionts can affect these hormonal pathways by either producing phytohormones themselves or modulating the host’s biosynthesis and signaling networks [20]. For example, increased auxin production has been correlated with enhanced root architecture and biomass accumulation, while ABA-related pathways are essential for mediating responses to osmotic stress and water deficit [21]. In contrast, beneficial symbioses are often linked to the suppression of defense-related hormone pathways, such as those involving ethylene and jasmonic acid, indicating a trade-off between growth and defense that promotes symbiotic compatibility [22]. This hormone-mediated reprogramming enables plants to optimize resource allocation and adapt more effectively to environmental challenges, among several other processes.

In parallel with hormonal regulation, plant responses to salinity stress are governed by intricate gene regulatory networks that involve transcription factors, signaling kinases, and downstream effectors. Key regulatory components include the salt overly sensitive (SOS) pathway, which maintains ion homeostasis through the extrusion of Na^+^ and tonoplast Na^+^/H^+^ exchangers that sequester toxic ions into vacuoles [6]. Furthermore, transcription factor families such as basic leucine zipper (bZIP), NAM, ATAF1/2, and CUC2 (NAC), dehydration-responsive element-binding (DREB), myeloblastosis (MYB), and basic helix-loop-helix (bHLH) are crucial in orchestrating stress-responsive gene expression [23]. ABA-dependent signaling pathways are particularly significant, as they integrate environmental cues with transcriptional responses to regulate stomatal closure, osmolyte accumulation, and stress-responsive gene expression [24]. Emerging evidence indicates that beneficial fungi can modulate these regulatory networks by either activating stress-responsive pathways or attenuating stress perception, thereby enhancing plant tolerance [25]. Recent studies have identified core regulatory modules involving ABA-responsive transcription factors and osmotic stress-related genes that are commonly induced in fungus-associated plants under salinity or osmotic stress [26,27]. These shared molecular signatures suggest that distinct symbiotic partners may converge on similar regulatory frameworks to achieve enhanced stress resilience.

Despite significant advancements, the molecular mechanisms by which fungi enhance salinity tolerance remain inadequately understood, particularly in terms of the integration of hormonal signaling and transcriptional regulation. Furthermore, while numerous studies have focused on well-characterized fungal genera, the functional potential of less-studied taxa remains largely unexplored. In this context, identifying and characterizing novel endophytic fungi from stress-adapted environments presents valuable opportunities to uncover new mechanisms of plant stress tolerance.

In the present study, we isolated and characterized root-associated fungal endophytes from the roots of *Oryza sativa* cv. Puntal cultivated in the marshes of the Guadalquivir River in southern Spain. We identified a previously uncharacterized strain, *Reticulascus* sp. S5, which significantly enhances plant growth under salinity stress conditions. Utilizing *Arabidopsis thaliana* as a non-native model system to test for a broader applicability of the fungus in sustainable agriculture, we combined phenotypic analyses with comprehensive transcriptomic profiling to elucidate the molecular basis of this beneficial interaction. Additionally, we compared the responses induced by *Reticulascus* sp. S5 with those elicited by a previously described salt stress tolerance conferring *Fusarium* strain [26] to explore potential convergence and divergence in symbiotic mechanisms. Both fungi, *Fusarium* sp. strain K-23 and *Reticulascus* sp. S5, share a common biomass promoting effect on plant shoots under salt stress conditions. Our findings provide new insights into how habitat-adapted fungal endosymbionts modulate plant hormone pathways and stress regulatory networks to enhance plant performance under salinity stress, thereby contributing to a deeper understanding of plant–microbe interactions and their potential applications in sustainable agriculture.

## 2. Results

### 2.1. Fungi Collection

**Figure 1:**
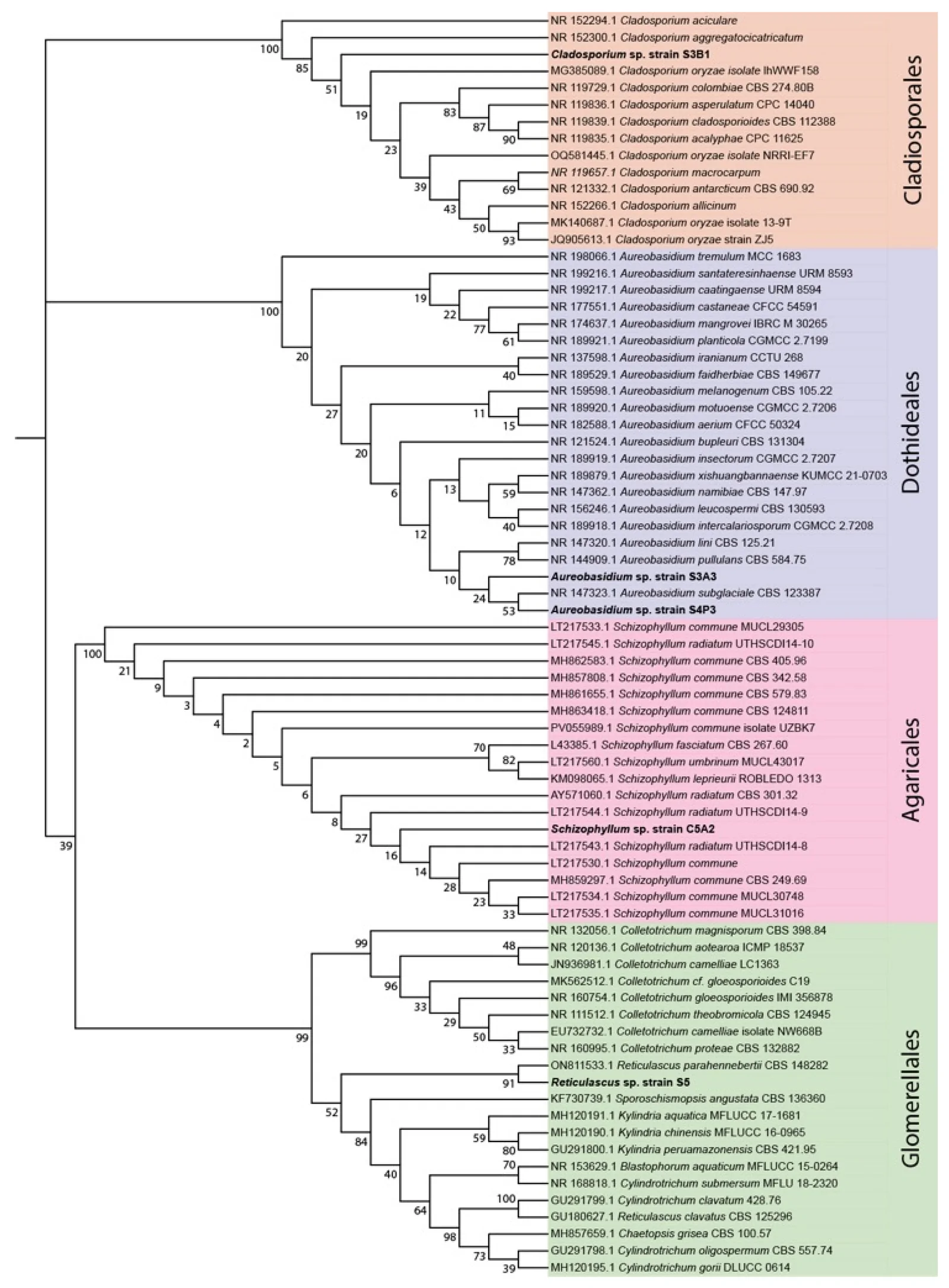
Evolutionary relationship of 75 fungal taxa. The evolutionary relationship of the studied taxa was inferred using the neighbor-joining method. The percentage of replicate trees in which the associated fungal taxa clustered together in the bootstrap test (10,000 replicates) are shown next to the branches. The phylogenetic tree includes 70 taxa obtained from the NCBI database and the 5 investigated isolates given in bold letters.

Initially, root endophytes from *Oryza sativa* cv. Puntal, harvested in the marshes of the Guadalquivir River, were isolated. Following multiple transfers to fresh potato dextrose agar (PDA) plates, a collection of 38 isolates was established. These isolates were subsequently cultivated axenically on PDA medium with progressively increasing concentrations of NaCl in the range between 200 mM and 1 M. Among the isolates tested, more than 10 demonstrated significant tolerance to NaCl and were therefore retained in the collection. To evaluate the plant growth-promoting effects of these isolates under salt stress conditions, they were co-cultivated with the non-native host plant *Arabidopsis thaliana* in vitro under control conditions and two distinct salt stress conditions (50 mM and 75 mM NaCl). Among the isolates tested, five demonstrated a significant plant growth-promoting effect on Arabidopsis, which suggested a conserved mode of action. Motivated by the beneficial effects on plant growth and stress tolerance observed under salt stress conditions, genomic DNA was extracted, and their ITS regions were amplified using PCR. The resulting DNA fragments underwent Sanger sequencing. The sequences obtained were subsequently compared with the 3.8 million publicly available ITS sequences in the UNITE Fungal ITS database [28] and the approximately 20,000 entries in the fungal ITS RNA RefSeq Targeted Loci (RTL) project database available at the National Center for Biotechnology Information (NCBI). A phylogenetic analysis was then conducted, revealing that from the five isolates, one belonged to the phylum Basidiomycota (Schyzophyllum) and four to the phylum Ascomycota (Cladosporium, Aureobasidium, Reticulascus). In summary, our collection comprised one *Cladosporium* sp., two *Aureobasidium* spp., one *Schizophyllum* sp., and one *Reticulascus* sp. strain (Figure 1).

The isolate, which is classified within the order Glomerellales [29], has garnered our specific interest. Numerous members of this order, such as various *Colletotrichium* strains including *Colletotrichium acutatum* and *Colletotrichium graminicola*, are well-documented plant pathogens known to cause substantial economic losses in agriculture [30,31]. Conversely, *Colletotrichium tofieldiae* has been identified as a highly effective fungal endophyte that considerably promotes the growth of its host plants [11]. The Reticulascaceae family, however, remains relatively understudied [32]. Members of this family are characterized by a saprophytic lifestyle, inhabiting dead wood and bark in both terrestrial and freshwater environments. Our isolate, *Reticulascus* sp. strain S5, belongs to the genus Reticulascus, which represents the sexual phase (teleomorph) of the fungus whose asexual phase (anamorph) is classified within *Cylindrotrichum*, which also appears in the phylogenetic tree presented in Figure 1. The strain most closely related to our S5 isolate is *Reticulascus parahennebertii*. This strain has been recently identified as a saprophyte of the dead culm of *Juncus inflexus* [33].

To date, no *Reticulascus* strain has been identified as a plant growth-promoting root-colonizing fungal endophyte capable of enhancing salinity stress tolerance in plants. Consequently, the *Reticulascus* sp. strain S5 was selected from our collection for further investigation.

### 2.2. Plant Growth Promoting Effect of Reticulascus sp. Strain S5

**Figure 2:**
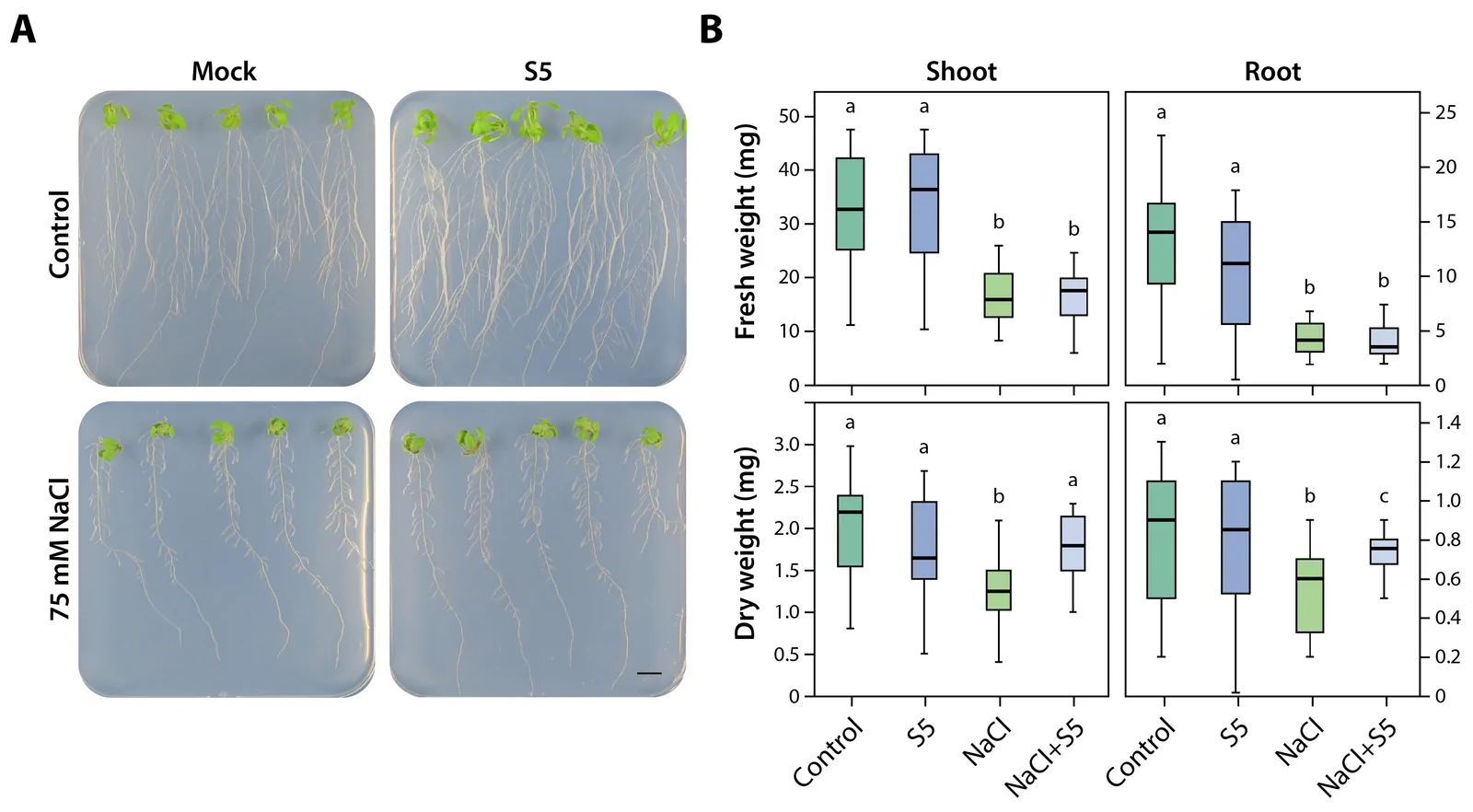
Phenotypic analysis of the growth-promoting effect of *Reticulascus* sp. strain S5 on *A. thaliana* seedlings in the presence and absence of salt stress. Scalebar: 10 mm. (**A**) Phenotype of Arabidopsis seedlings that were either mock-treated or inoculated with 20 μL of a solution containing 1 × 10^5^* Reticulascus* sp. S5 spores mL^−1^ at 10 dpi. (**B**) Fresh and dry weight analysis of the shoots and roots of mock (Control) and S5-inoculated (S5) plants, as well as mock (NaCl) and S5-inoculated (NaCl+S5) plants, respectively, on plates containing 75 mM NaCl. The box plots show the median, quartiles, and extremes of the compared datasets (*n* = 25). For the statistical analysis, one-way ANOVA with a post hoc Tukey–Kramer test was employed. Different letters indicate significant differences between means.

Before testing the plant growth promoting effect of S5 on *A. thaliana*, the colonization of the roots was confirmed by re-isolation experiments. As illustrated in Supplementary Figure S1, the fungus was able to enter and inhabit Arabidopsis roots without causing harm to the plant. The emergence of hyphae from the open end of the root fragment indicated that the hyphae, which were safeguarded within the apoplast, inside the root, were able to regrow and extend out of the root, confirming an endophytic lifestyle not only in the original host plant, rice, but also in Arabidopsis. Next, the impact of S5 on wild-type *A. thaliana* plants was examined. Seven-day-old Arabidopsis seedlings were co-cultivated with the fungus and compared to mock-treated control plants, both in the presence and absence of salt in the growth medium. As depicted in Figure 2, fungal inoculation led to a significant increase in plant biomass of the root and shoot tissues under salt stress conditions. Conversely, in the absence of salt stress, the fungus-inoculation did not result in any significant difference in biomass production, although the root system, particularly the length of the lateral roots, appeared to be enhanced.

A closer inspection of the root system architecture under salt stress provided evidence for 15.7% longer primary roots (*p* = 0.001) of the S4-inoculated plants compared to the mock control plants. In contrast, lateral root density appeared to be reduced by 18.5% (*p* = 0.028) when compared to the salt-treated control plants. The obtained results suggest a fungus-mediated rewiring of the plant growth program under salt stress conditions, resulting in increased overall biomass and an altered root system architecture.

### 2.3. Global Transcriptomic Reprogramming of Arabidopsis Plants by Reticulascus sp. Strain S5

**Figure 3:**
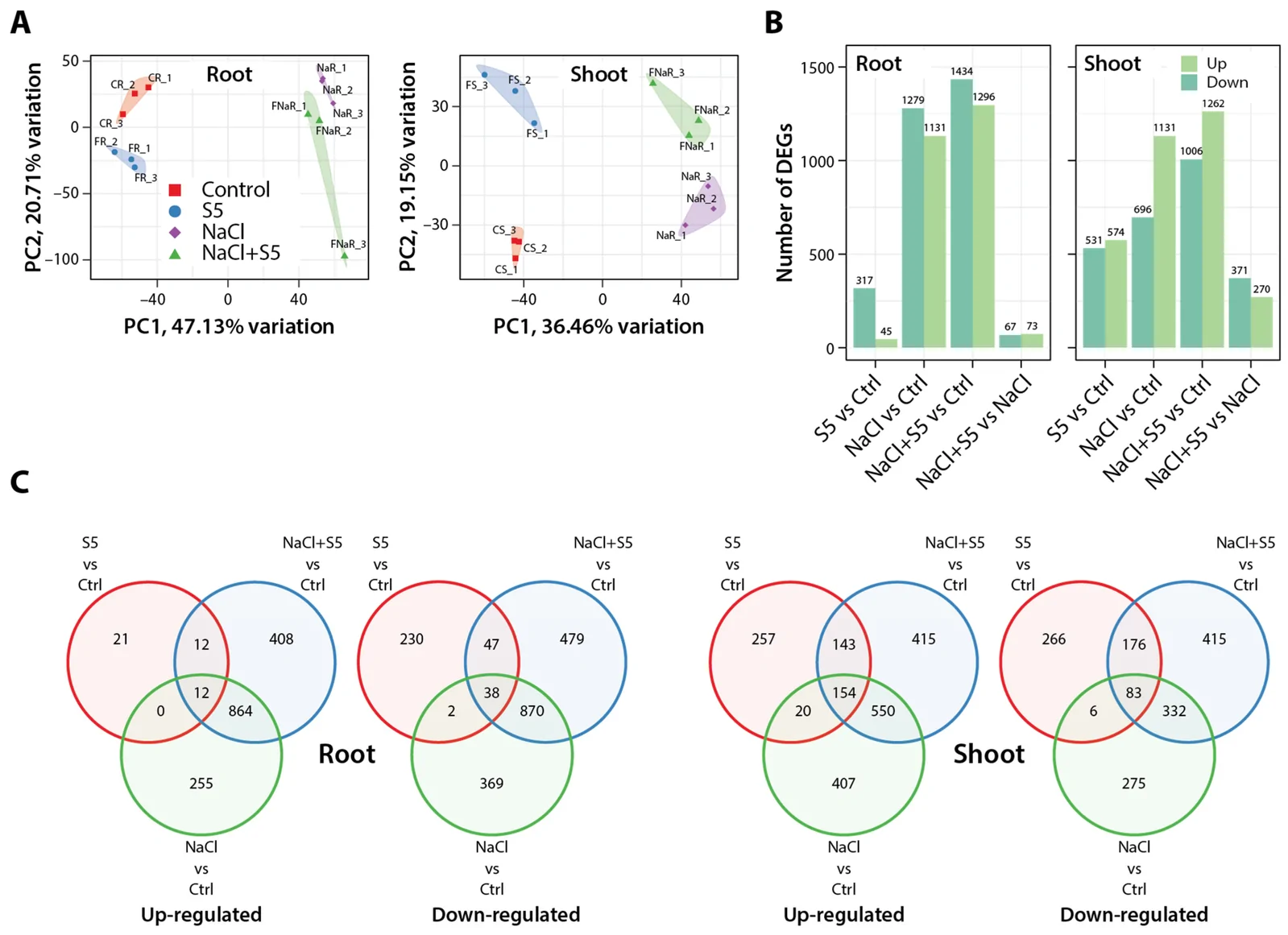
Transcriptional response of Arabidopsis seedlings to *Reticulascus* sp. strain S5 inoculation and salinity stress. (**A**) Principal component analysis (PCA) of transcriptome-wide normalized gene expression counts in the roots and shoots assessing the difference in mock (Control) and S5 (S5) inoculation wild-type Col-0 seedlings in the presence and absence of 75 mM NaCl in the medium (NaCl and NaCl+S5). Each different shape represents a sample; colors and symbols refer to inoculation status and stress condition. The x-axis denotes the PC1; y-axis denotes values for PC2. (**B**) Separate DEGs statistics for the mock and S5-inoculated Col-0 root and shoot tissues in the presence or absence of 75 mM NaCl at 10 dpi. (**C**) Venn diagram analysis for the shoot and root samples showing the number of DEGs shared between the mock-inoculated control plants (Ctrl) and similarly grown plants under salinity stress (NaCl). A second batch of Arabidopsis seedlings was inoculated with the fungus under control conditions (S5) versus S5-inoculated plants grown under salinity stress conditions (NaCl+S5) at 10 dpi. Induced (upregulated) and repressed (downregulated) DEGs were separately evaluated.

To obtain a comprehensive understanding of the transcriptomic responses to fungal exposure under both control and salt stress conditions, a full factorial mRNA sequencing (RNA-Seq) experiment was conducted. This experiment encompassed four distinct conditions: Control (mock-treated, no salt stress), NaCl (mock-treated, salt stress), S5 (fungus-inoculated, no salt stress), and NaCl+S5 (fungus-inoculated, salt stress). For each condition, three independent biological replicates were analyzed. Root and shoot tissues were examined separately (Supplementary Table S1). Principal component analysis (PCA) demonstrated a clear differentiation among the tested groups for both the root and shoot samples. The first two principal components (PCs) accounted for 67.9% and 55.6% of the variance in the root and shoot samples, respectively. Notably, in both tissues, PC1 was closely associated with salinity stress, whereas PC2 appeared to be linked to the inoculation condition (Figure 3A). The individual replicates demonstrated effective clustering within each experimental condition and tissue type, thereby distinguishing the various sample groups into distinct transcriptional profiles. This observation indicated a high level of experimental reproducibility. Analysis of the general expression profiles across various conditions revealed an unexpectedly stronger response to the fungus (S5 versus Ctrl) in the shoots compared to the roots (Figure 3B). In contrast, the responses to salt (NaCl versus Ctrl) aligned with previously published data and did not present any unexpected findings [14,26]. Interestingly, the number of differentially expressed genes (DEGs) in response to S5 inoculation was consistently lower under salt conditions (NaCl+S5 versus NaCl) compared to S5-inoculated plants grown without salt (S5 versus Ctrl). A detailed analysis contrasting the DEGs in the two groups revealed minimal overlap, with no more than 13 and 241 genes in the roots and shoots, respectively (Supplementary Figure S2). However, the 241 DEGs in the intersection between S5 versus Ctrl and NaCl+S5 versus NaCl in the shoots showed enrichment in the Kyoto Encyclopedia of Genes and Genomes (KEGG) pathways related to glucosinolate biosynthesis (Path:ath00966) and 2-oxocarboxylic acid metabolism (Path:ath01210), suggesting alterations of biotic stress responses. Furthermore, a subsequent Gene Ontology (GO) enrichment analysis highlighted terms related to response to abiotic stimulus (GO: 0009628), response to stress (GO: 0006950), and glucosinolate biosynthetic processes (GO: 0019761), which also points toward an alteration of biotic stress responses.

**Figure 4:**
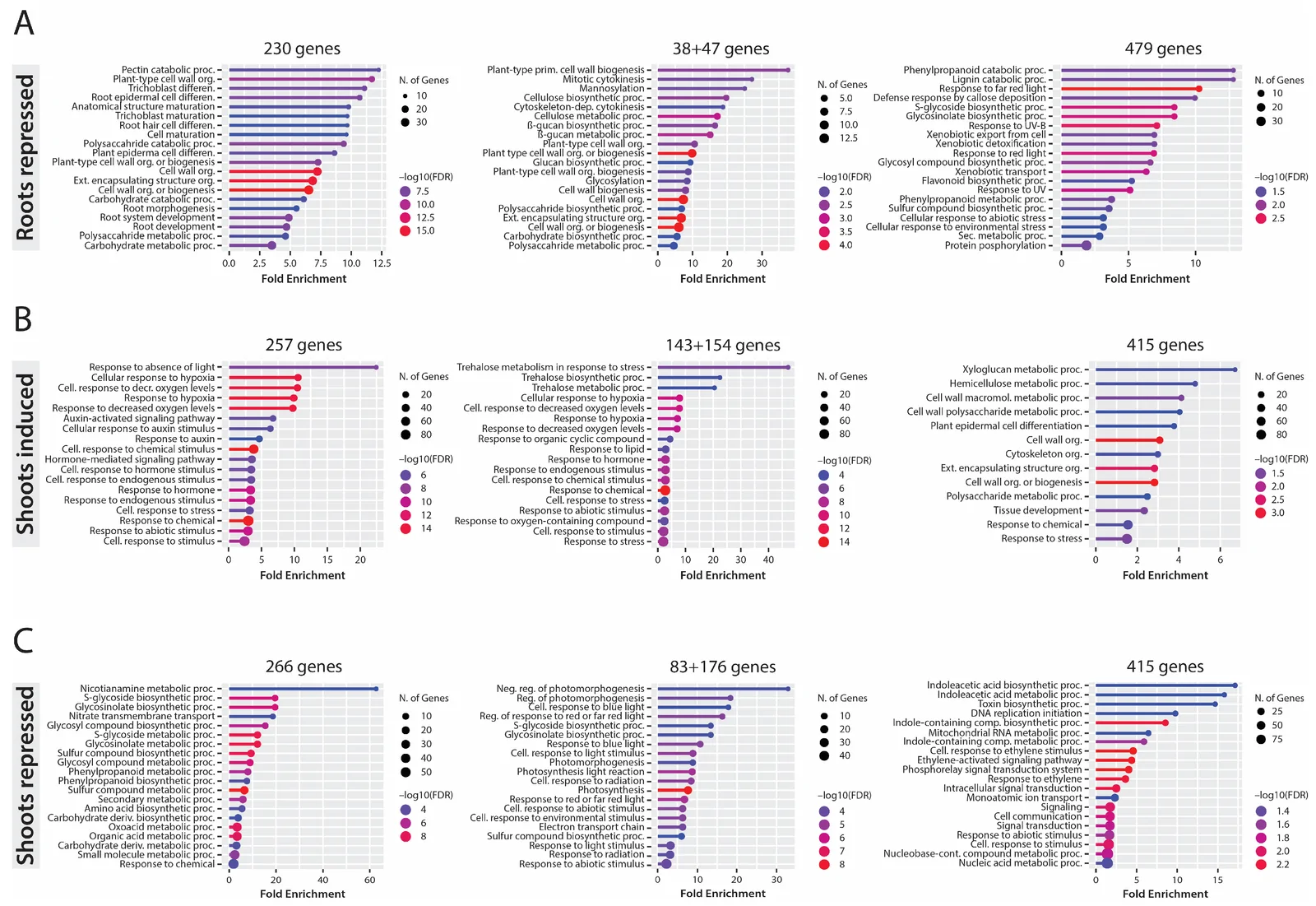
Gene enrichment analysis of Arabidopsis roots and shoots responding to an inoculation with *Reticulascus* sp. strain S5 and salinity stress. (**A**) GO enrichment analysis of the 230 repressed DEGs in the S5 versus Ctrl comparison (**left**), the 479 genes exclusively repressed in the NaCl+S5 versus Ctrl comparison (**right**), and the 85 genes in the intersections of the two previous groups in the root samples. GO enrichment analysis of the induced (**B**) and repressed genes (**C**) in the corresponding groups (S5 versus Ctrl, intersections between S5 versus Ctrl and NaCl+ S5 versus Ctrl, NaCl+S5 versus Ctrl, from left to right) in the shoot samples. Each circle in the figure represents a distinct GO term. The circle size indicates the number of genes enriched in the corresponding GO term. The significance of the observed gene enrichment is represented by a color gradient referring to the -log10(FDR) value.

To gain a comprehensive understanding of the relationships among the DEGs, Venn diagram analyses were performed to assess both induced and repressed genes in the root and shoot samples separately (Figure 3C). This analysis specifically targeted distinct gene groups responding to fungal inoculation in the absence (S5 versus Ctrl) and presence of salt stress (NaCl+S5 versus Ctrl), as well as DEGs located at the intersections of these two groups. The identified gene groups were subsequently subjected to KEGG pathway and GO term enrichment analysis. The full GO term and KEGG pathway enrichment analysis is included in Supplementary Table S2. Although the 21 and 408 genes induced in the roots under the test conditions, along with the 24 genes at the intersections, did not exhibit significant enrichment in KEGG pathways or GO terms, the analysis of the repressed DEGs revealed several notable biological processes influenced by the fungus. As illustrated in Figure 4A, the GO analysis of the 230 repressed DEGs in S5-inoculated plants without salt stress (S5 versus Ctrl) highlighted the repression of genes categorized under GO term classifications related to cell wall organization (GO:0022622), root development (GO:0048364), and root hair initiation and development, including terms such as trichoblast differentiation (GO:0010054), trichoblast maturation (GO:0048764), and root hair cell differentiation (GO:0048765). Notably, it revealed the repression of the root hair initiation and development master regulator genes *RHD6* and *RSL4*. Conversely, the 85 genes at the intersection between S5 versus Ctrl and NaCl+S5 versus Ctrl exhibited enrichment in cell wall organization-related terms (GO:0071555), including a pronounced repression of cellulose biosynthesis-related genes (GO:0030244), such as *CSLB2*, *CSLB3*, *CSLB5*, and *CSLB6*. Meanwhile, the 479 repressed genes in the NaCl+S5 versus Ctrl comparison highlighted an enrichment of defense and stress-related terms, such as glucosinolate biosynthetic processes (GO:0019761) and cellular response to abiotic stimulus (GO:0071214).

This suggests that the fungus appears to reduce root hair growth under control conditions, while secondary cell wall biosynthesis and plant stress responses seem to be diminished by the fungus under salinity conditions. The analysis of gene groups induced in the shoots (Figure 4B) revealed significant insights into the impact of the fungus on shoot development. Among the 257 genes that were upregulated under control conditions when comparing the S5-inoculated plants with the mock-treated plants, there was a notable enrichment of auxin-related GO terms. These included classifications such as the regulation of hormone levels (GO:0010817), auxin-activated signaling pathway (GO:0009734), and response to auxin (GO:0009733). Prominent genes within this group comprised auxin biosynthesis genes *YUC2*, *YUC5*, and *YUC8*, alongside several Aux/IAA genes and small auxin upregulated RNA genes, including *IAA1*, *IAA2*, *IAA5*, *IAA6*, *IAA19*, *IAA32*, *SAUR19*, *SAUR22*, *SAUR23*, *SAUR26*, and *SAUR28*. The findings strongly suggest an increase in auxin content within the shoots of the fungus-inoculated plants. Visual inspection indicated that the leaf sizes of the inoculated plants under control conditions were slightly larger. However, measurements of both fresh and dry weight did not reveal significant differences. In the analysis of GO terms enriched at the intersection of the control versus salt stress comparison, additional genes associated with hormone response terms were identified. These included response to auxin (GO:0009733), response to hormone (GO:0009725), and cellular response to hormone stimulus (GO:0032870). These categories contained further auxin-related genes such as *YUC9*, the GRETCHEN HAGEN 3 (GH3) acyl acid amido synthetase genes *WES1*|*GH3.5* and *DFL1*|*GH3.6*, which are involved in auxin conjugation, as well as the auxin exporter gene *PIN3*. Notably, the analysis also revealed the induction of genes associated with other stress-related hormonal responses, including those involved in ABA biosynthesis, signaling, and response, such as *NCED3*, *AFP1*, *AFP3*, *HAI1*, *LTI65*|*RD29B*, and *EEL*|*bZIP12*, which are linked to general abiotic stress and water deprivation stress responses. Additionally, several jasmonic acid-linked genes, including *JAZ3*, *JAZ7*, *BT2*, and *BT4*, were identified. The analysis of the 415 genes uniquely induced in the NaCl+S5 versus Ctrl comparison revealed a significant enrichment of GO terms related to cell wall biogenesis and organization. Notably, these terms include the classifications cell wall organization or biogenesis (GO:0071554) and cell wall macromolecule metabolic process (GO:0044036). A substantial number of XYLOGLUCAN ENDO-TRANSGLYCOSYLASE/HYDROLASE (XTH) genes, such as *XTH11*, *XTH15*, *XTH16*, and *XTH18*, were identified. These genes play a crucial role in loosening the rigid cellulose–xyloglucan network, thereby facilitating remodeling of the primary cell wall through their enzymatic activity in hemicellulose and xyloglucan metabolic processes.

Similar to the case of repressed genes in the roots, the analysis of identified repressed genes in the shoots strongly indicates a reduction in plant defense and stress responses (Figure 4C). The 266 genes repressed in the S5 versus Ctrl comparison demonstrate the downregulation of defense compound production, such as glucosinolates, as evidenced by the significant enrichment of genes in the GO terms glucosinolate biosynthetic process (GO:0019761) and phenylpropanoid biosynthetic process (GO:0009699). A similar pattern, with a more pronounced abiotic stress component induced by additional salt stress, was observed in the 257 genes within the overlapping segments. The GO classification of the 415 genes exclusively repressed in the NaCl+S5 versus Ctrl condition further substantiates the systematic repression of plant defense responses. Notably, genes such as *SUR1*, *SUR2*|*CYP83B1*, and *WRKY70*, encompassed in the GO term indole-containing compound biosynthetic process (GO:0042435), were significantly underrepresented. Conversely, this group also showed an underrepresentation of ethylene-related biological processes, including cellular response to ethylene stimulus (GO:0071369), response to ethylene (GO:0009723), and ethylene-activated signaling pathway (GO:0009873). Ethylene is recognized as a central hub in environmental and metabolic stress responses, coordinating defense mechanisms and dynamically balancing plant growth to facilitate adaptation to adverse environmental conditions [34]. Hence, it must be concluded that these adaptation processes are not triggered at the same level in fungus inoculated plants, possibly because of a lower experienced abiotic stress level.

### 2.4. Comparison of the Transcriptional Responses of Arabidopsis to the Inoculation with Reticulascus sp. Strain S5 or Fusarium sp. Strain K-23

**Figure 5:**
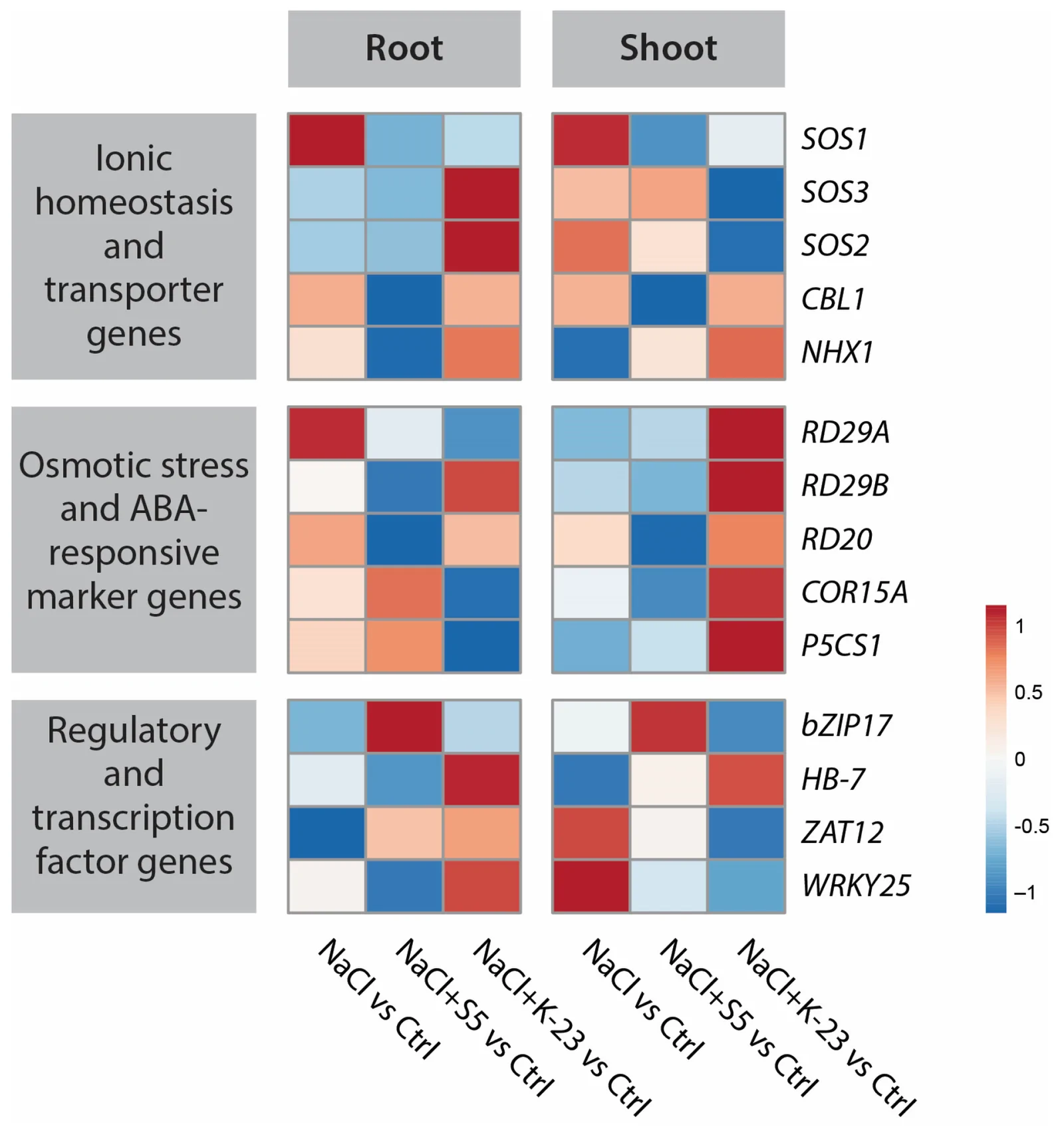
Heatmap illustrating the transcriptional responses of 14 salt stress marker genes under salinity stress conditions, both in the absence and presence of *Reticulascus* sp. strain S5 and *Fusarium* sp. strain K-23. These genes were categorized into three functional groups: those related to ion homeostasis, osmotic stress and ABA responsiveness, and gene regulation.

In a prior study [26], we characterized a *Fusarium* strain isolated from the Himalayas near Kargil in the Jammu and Kashmïr region. Analogous to the *Reticulascus* sp. S5 strain, the *Fusarium* sp. K-23 strain enhanced the tolerance of *A. thaliana* to salt stress. Under saline conditions, K-23-inoculated plants exhibited increased total biomass, shoot fresh weight, and root system length compared with the non-inoculated controls. The fungus significantly enhanced root hair development under control conditions and effectively mitigated the salt-induced inhibition of root hair growth. This led to increased root hair density and elongation under saline conditions. This prompted us to compare the transcriptional responses of Arabidopsis plants inoculated with the two fungi. In an initial approach, the expression profiles of 14 salt stress marker genes were studied (Figure 5).

Evidence from the profiling indicated that both fungi induce transcriptional repression of the Na+/H+ antiporter gene *SOS1*. Additionally, there was a common regulatory effect on the transcription factor genes *HB-7*, which was induced, and *WRKY25* and *ZAT12*, which were repressed. However, the majority of the other markers exhibited a less definitive pattern, demanding a more extensive transcriptional comparison.

**Figure 6:**
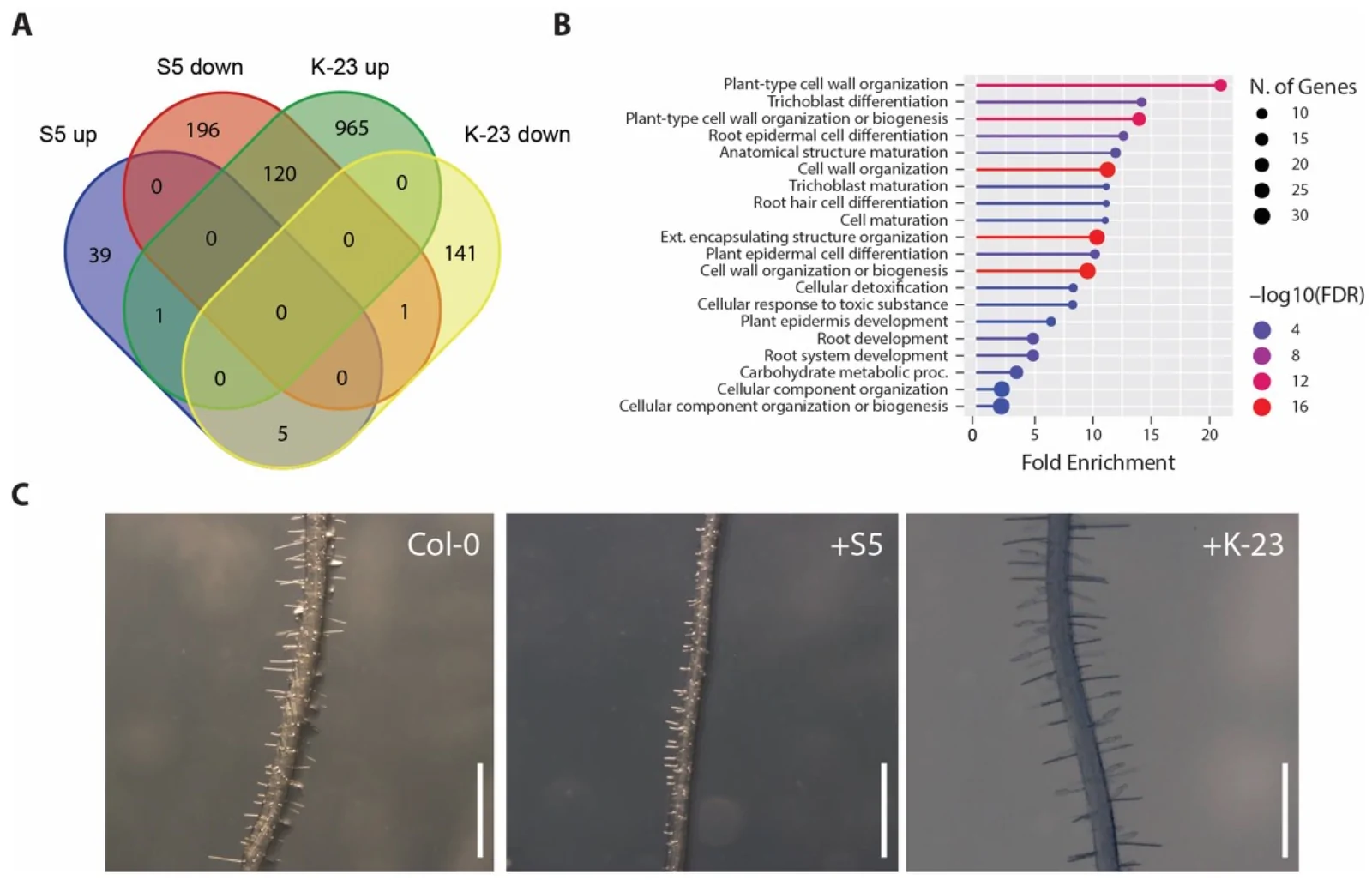
Comparative analysis of the transcriptomic effects of infections by *Reticulascus* sp. strain S5 and *Fusarium* sp. strain K-23 on Arabidopsis roots. (**A**) Venn diagram showing the numbers of DEGs induced (up) and repressed (down) in the S5- and K-23 inoculated Arabidopsis roots, respectively, under control conditions. (**B**) GO enrichment analysis of the 120 DEGs in the intersection of repressed genes in the S5-inoculated plant roots and induced genes in the K-23-inoculated plant roots. Each circle in the figure represents a distinct GO term. The circle size indicates the number of genes enriched in the corresponding GO term. The significance of the observed gene enrichment is represented by a color gradient referring to the -log10(FDR) value. (**C**) Representative images of root hairs from Arabidopsis control seedlings (Col-0) grown for 7 days on 0.5 × MS and another 7 days on PNM minimal medium, and similarly grown infected seedlings. Scalebar: 400 µm.

Unlike S5, which suppressed genes related to root hair initiation and development, K-23 was previously observed to promote root hair growth even under salt stress [26]. This sustained root hair growth was demonstrated to be crucial for the plant growth-promoting effect observed under salinity stress. This seemingly contradictory mechanism led us to first compare the molecular modules influenced by the two strains in their host plants to better understand how habitat-driven co-evolution can result in similar phenotypes, albeit potentially through different molecular pathways. As illustrated in Figure 6, the comparison of DEGs in S5 versus the control and K-23 versus the control (GEO dataset GSE260960) revealed that 35.2% of the 361 DEGs in the S5-inoculated plants were shared with genes responsive to K-23 inoculation. However, the 120 genes that represented the majority of the shared DEGs were found in the intersection of the genes repressed by S5 but induced by K-23. A detailed examination (Figure 6B) revealed that these genes were associated with root developmental processes and root hair growth-related GO terms.

The subsequent microscopic examination of root hairs on primary roots of both control Col-0 plants and those inoculated with S5 and K-23, as depicted in Figure 6C, confirmed the transcriptomics data at the phenotypic level. While the inoculation by the two studied fungal strains resulted in distinct phenotypes concerning root hair growth, the phenotypic impact on shoots was observed to be similar.

**Figure 7:**
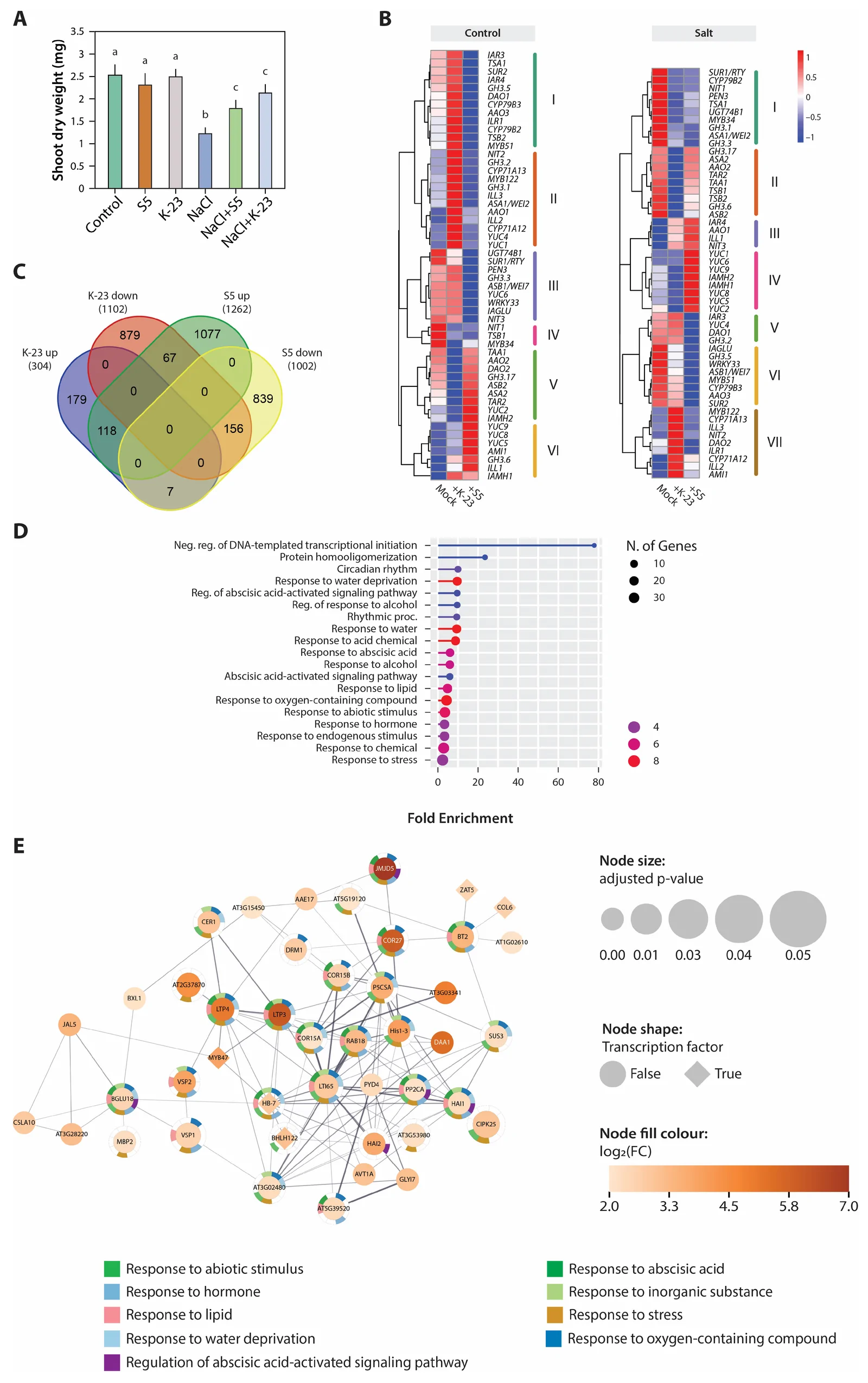
Phenotypic and transcriptomics analysis of the growth-promoting effects of *Fusarium* sp. K-23 and *Reticulascus* sp. S5-inoculations on Arabidopsis shoots under control and salt stress conditions. (**A**) Shoot dry weights of Arabidopsis plants grown under distinct conditions. The bar plots show the means ± their standard error of means (*n* = 20). Two-way ANOVA with a post hoc Tukey–Kramer test was used. Different letters indicate significant differences between means (*p* ≤ 0.05). (**B**) Hierarchical clustering heatmap of normalized read counts of 55 genes related with auxin-involving biological processes in the mock-treated control plants (Mock), K-23-inoculated plants (+K-23), and S5-inoculated plants (+S5) in the absence (Control) and presence of 75 mM NaCl (salt). (**C**) Venn diagram analysis for shoot samples showing the number of DEGs shared between the K-23-inoculated and S5-inoculated plants grown under salt stress conditions at 10 days post infection. Induced (upregulated) and repressed (downregulated) DEGs were separately evaluated. (**D**) GO enrichment analysis of the 118 induced DEGs in the K-23 versus S5 comparison in the shoots of Arabidopsis plants grown under salt stress. Each circle in the figure represents a distinct GO term. The circle size indicates the number of genes enriched in the corresponding GO term. The significance of the observed gene enrichment is represented by a color gradient referring to the -log10(FDR) value. (**E**) Functional network analysis of the identified 118 DEGs shared between the shoots of the K-23 and S5-inoculated plants grown under salinity stress conditions. The color of the ring portions around the spheres indicate the GO terms to which the different genes are associated.

Inoculation of Arabidopsis plants with both strains led to a significant increase in biomass formation compared to mock-treated plants under identical conditions (Figure 7A). Considering the significant enrichment of auxin-related processes in S5-inoculation shoots, the similar biomass production under stress motivated us to compare perform a hierarchical clustering of genes associated with the metabolism of auxin and auxin-derived plant defense compounds, including glucosinolates and camalexin. As depicted in Figure 7B, transcript abundance has been clustered for control and salt stress condition. Under both control and salt stress conditions, marked differences were observed between inoculations with the two strains and the mock-infected plants. Notably, K-23-inoculated plants exhibited greater similarity to the mock-treated control plants. Under control conditions, the transcript abundance of auxin biosynthesis-related genes in cluster VI appeared higher in fungus-inoculated samples compared to the mock control. Additionally, in cluster IV, the abundance of genes such as *MYB34* was reduced by both fungal inoculations. Under salt stress conditions, more correlations were identified between the fungus-inoculated samples and the mock control. In cluster I and VI, groups of less abundant genes, primarily associated with defense compound-related biosynthetic processes, were identified in the K-23- and S5-inoculated samples. Conversely, in cluster IV, an increased abundance of auxin-conjugate hydrolyzation-related genes was observed.

Next, we decided to compare the DEGs identified in the shoot samples under the NaCl+S5 versus control and NaCl+K-23 versus control conditions. As illustrated in Figure 7C, overlapping groups of induced and repressed genes were identified in the fungus-inoculated samples. The analysis of the 156 commonly repressed genes revealed an enrichment of GO terms associated with responses to light stimulus and radiation (Supplementary Table S3). Furthermore, the classification of the 118 commonly induced genes across the two compared datasets indicated an enrichment in GO terms predominantly related to ABA-dependent processes, including responses to water, water deprivation, and ABA. This finding led us to conclude that the fungus inoculations triggered a reprogramming of the abiotic stress response, suggesting a more robust stress response. To gain a more comprehensive understanding of the putative regulatory network orchestrating this reprogramming, we conducted a functional network analysis using the 118 commonly induced genes as the query input. As illustrated in Figure 7E, the analysis identified a core regulatory framework consisting of three interconnected transcription factors: *MYB47*, *HB-7*, and *bHLH122*. These factors are closely associated with several osmotic stress regulatory elements, including *P5CSA*, which encodes Δ^1^-pyrroline-5-carboxylate synthase A, a rate-limiting enzyme in proline biosynthesis. Additionally, *HAI1*, *HAI2*, and *PP2CA* encode protein phosphatases that serve as negative regulators of Snf1-related protein kinase1 (SnRK1) signalling. Concurrently, *HB-7* is functionally linked to *BGLU18*, a gene encoding β-*d*-glucopyranosyl abscisate β-glucosidase, which catalyses the hydrolysis of ABA glucose esters.

## 3. Discussion

Salt stress significantly limits plant growth and agricultural productivity by inducing osmotic and ionic stresses that disrupt cellular homeostasis, inhibit photosynthesis, and decrease biomass accumulation and yield [1,3]. In the context of current climate change, which is associated with rising sea levels, rice production in Spain is also impacted. Spain is the second largest rice-producing country in Europe. The marshes of the Guadalquivir River, known as the ‘Marismas del Guadalquivir’, cover approximately 40,000 hectares and represent the most significant rice-producing region in Spain, accounting for approximately 39% of the country’s rice cultivation [35]. The region is increasingly affected by tidal influences, resulting in the intrusion of seawater into the marshes and its subsequent mixing with freshwater. This phenomenon adversely affects rice grain production, which is typically sensitive to salinity [36]. However, approximately 75% of the rice cultivated in the marshes belongs to the *Oryza sativa* cultivar (cv.) Puntal. This cultivar is of Australian origin (known as ‘Doongara’ in Australia) and belongs to the *Indica* (long grain) subgroup and demonstrates considerable adaptability to fluctuating salinity levels. Recent publications have indicated that beneficial plant-colonizing fungi can enhance their host plants’ adaptation to adverse environmental conditions, including salinity [37]. Consequently, we hypothesized that Puntal rice plants, due to habitat-adapted symbiosis, may host a mycobiome that assists in tolerating elevated levels of salt stress. While there has been a report on bacteria isolated from rice plants in this area [38], there remained a knowledge gap regarding the mycobiome of these plants. To address this gap, fungal endophytes were isolated from the roots of Puntal plants collected from paddy fields on two farms near the village of La Puebla del Río, located south of Seville. As expected, a substantial number of fungal endophytes were isolated from the roots. Among these, five strains were selected for further study (Figure 1) due to their demonstrated strong salt tolerance in in vitro analyses. Notably, these strains also conferred significant salt tolerance to the non-host plant *A. thaliana*, indicating a highly conserved mode of action rather than a highly specialized symbiosis between strongly adapted partner organisms, which is of particular interest considering a possibly broader application in agriculture.

This study initially concentrated on the fungal strain *Reticulascus* sp. strain S5, as this root-colonizing fungal endophyte exhibited a notable growth-promoting effect on the roots and shoots of inoculated plants under conditions of salt stress (Figure 2, Supplementary Figure S1). At the molecular level, salinity triggers rapid osmotic stress signals, leading to stomatal closure and a decrease in carbon assimilation. Subsequently, there is an accumulation of toxic Na^+^ and Cl^−^ ions, which disrupt essential biochemical processes. Plants have developed complex tolerance mechanisms facilitated by coordinated signaling networks, notably the SOS pathway [6]. Within this pathway, the Ca^2+^ sensor SOS3 and kinase SOS2 activate the plasma membrane Na^+^/H^+^ antiporter SOS1, which maintains ion homeostasis by extruding Na^+^ from cells. Furthermore, the compartmentalization of Na^+^ into vacuoles via tonoplast Na^+^/H^+^ exchangers (NHX proteins) mitigates cytosolic toxicity [7]. Osmotic adjustment is accomplished through the synthesis of compatible solutes, such as proline, glycine betaine, and soluble sugars, which stabilize proteins and membranes while preserving turgor pressure. Salt stress also induces the production of ROS; plants counteract oxidative damage by activating antioxidant defense systems, including superoxide dismutase, catalase, and ascorbate peroxidase. Hormonal regulation, particularly through ABA, is crucial in modulating stress-responsive gene expression, ion transport, and stomatal conductance. Additionally, transcription factors such as DREB, NAC, and bZIP are involved in regulating downstream stress-responsive genes that enhance tolerance [4]. However, despite these adaptive mechanisms, prolonged or severe salinity can overwhelm plant defense systems, leading to impaired development and reduced crop yield. As a result, soil salinization represents a significant global threat to sustainable agriculture, particularly in irrigated and arid regions where salt accumulation is prevalent.

To explore the molecular mechanisms by which *Reticulascus* sp. strain S5 promotes plant growth under salinity stress, we employed a comprehensive transcriptomic analysis using RNA-Seq. Notably, our research demonstrated that the fungal inoculation significantly repressed *SOS1* expression (Figure 5), while other pathway components and the other seven NHX Na^+^/H^+^ antiporter genes demonstrated no significant alteration of their expression. The comprehensive analysis of RNA-Seq data revealed evidence for several distinct groups of biological processes that were either overrepresented or underrepresented in response to S5 inoculation, with and without the presence of salt in the growth medium (Figure 3 and Figure 4). In shoot tissues, we gathered evidence for a possible reprogramming of plant hormone-related processes, particularly those involving auxin and ABA metabolism, signaling, and response-related gene groups, which aligns with the observed increase in biomass production. It appears that cell wall loosening, facilitated by the induction of various *XTH* genes, is part of the response potentially involved in cell expansion. Furthermore, we identified a systematic repression of plant defense and senescence-related biological processes, evidenced by the repression of glucosinolate and phenylpropanoid biosynthesis pathways and ethylene signaling, respectively [20,21,22]. These observations, coupled with increased growth under stress conditions, suggest that the shoots of fungus-inoculated plants experience reduced abiotic stress, thereby facilitating enhanced growth. Concurrently, plants co-cultivated with S5 exhibited diminished defense mechanisms, likely promoting symbiosis with the fungus.

In roots, fungal inoculation intriguingly led to the suppression of genes associated with root hair growth. Under additional salt stress, we observed a notable underrepresentation of gene groups related to cell wall organization, cellulose biosynthesis, plant defense, and abiotic stress. These gene expression profiles, particularly the diminished expression of abiotic stress response-associated genes, imply that the fungus may either shield the host plant from salt stress or that the fungal inoculation itself induced differential gene expression. The root hair phenotype was of particular interest, as we recently documented another fungus, *Fusarium* sp. strain K-23, from the Himalayas that also enhanced salt tolerance, contingent upon the induction of root hair growth under both control and salt stress conditions [9]. This observation led us to compare the molecular responses elicited by these distinct fungal symbionts to elucidate how the evolution of habitat-adapted symbiosis might influence plant responses and whether similar responses are adopted to achieve comparable phenotypic outcomes in distinct biological settings [16,17]. The comparison of the root RNA-Seq data of Arabidopsis plants inoculated with the two fungi largely confirmed the expectations, as a considerable group of root hair development-related genes appeared to be induced in the K-23-inoculated plants while being repressed in the S5-inoculated plants. The distinct root hair phenotypes could also be corroborated experimentally (Figure 6).

These findings suggest that enhanced salinity tolerance is not necessarily associated with a unique developmental response in the root system. While *Fusarium* sp. K-23 promotes root hair formation, *Reticulascus* sp. S5 represses root hair-associated developmental pathways, yet both symbionts ultimately improve plant performance under saline conditions. This observation indicates that distinct fungal partners may employ alternative root-level strategies to achieve similar adaptive outcomes in the host plant.

Thus, the investigation into whether the transcriptional responses in the shoot samples exhibited greater comparability was of particular interest to us, given that a similar plant growth-promoting phenotype was observed in the aerial parts of plants inoculated with fungi under stress conditions (Figure 7). Although the auxin-related gene responses under the test conditions were not identical, certain similarities were identified, leading to the conclusion that both fungi are likely to stimulate plant growth by promoting auxin biosynthesis in the shoots. However, a more detailed analysis of the auxin effects needs to be conducted. Furthermore, the comparative analysis of the identified DEGs in the K-23 and S5-inoculated plants under salt stress revealed small groups of overlapping genes that appear to contribute to increased stress tolerance, and consequently, enhanced growth in the inoculated plants. A functional network analysis of the 118 shared genes induced by both fungi identified a core gene regulatory network centered around at least two transcription factor genes, *bHLH122* and *HB-7*. Notably, bHLH122 has been implicated in the regulation of drought and osmotic stress resistance in Arabidopsis and also contributes to the control of cellular ABA levels [22]. The homeodomain-leucine zipper (HD-Zip) class I transcription factor HB-7 is known to be involved in the regulation of water deficit responses [22,23,24]. Together, these transcription factors subsequently orchestrate the expression of several genes, including *HAI1*, *HAI2*, *PP2CA*, and *P5CSA*, which are crucial for fine-tuning ABA responses and controlling osmotic adjustment through the synthesis of osmoprotectant solutes, such as proline [25,26,27,28]. Overall, this suggests that the two fungi activate the same intrinsic response module in Arabidopsis plants under salt stress. The apparent convergence of two phylogenetically and ecologically distinct fungal symbionts on a common ABA-associated regulatory network is particularly interesting. Despite inducing markedly different transcriptional and developmental responses in the roots, both fungi activate a shared set of stress-responsive genes in the shoots. These results support the existence of conserved host regulatory modules that can be recruited by different beneficial microorganisms to enhance adaptation to saline environments. Interestingly, a similar observation was recently made when comparing the effect of three distinct fungal endophytes on the drought tolerance response of tomato plants [29].

Future research will face the challenge of further characterizing the role of the identified regulatory network in beneficial plant–fungus interactions. Additionally, elucidating the mechanisms of root-to-shoot communication within the investigated symbiotic relationships will be an exciting endeavor. Finally, understanding these mechanisms may facilitate the development of microbiome-based strategies to improve crop performance under increasing soil salinization. However, in order to achieve the latter goal, a broader group of crops needs to be tested with the fungi in the future.

## 4. Materials and Methods

### 4.1. Plant Material and Growth Conditions

In this work, we used *Arabidopsis thaliana* L. var. Col-0 (N1092) obtained from the Nottingham Arabidopsis Stock Center (NASC) (Nottingham, UK). Following surface sterilization of seeds using 70% (*v*/*v*) ethanol (2 min) and 2% (*w*/*v*) sodium hypochlorite (5 min), the seeds underwent stratification for 2 days at 4 °C. Next, the seeds were transferred to plates and cultivated sterilely on solidified 0.5 × MS medium supplemented with 1% (*w*/*v*) sucrose [39]. The plants were maintained in growth chambers under rigorously controlled environmental conditions, including a photoperiod of 16 h of light and 8 h of darkness, a constant temperature of 22 °C, and photosynthetically active radiation of 100–105 μmol photons m^−2^ s^−1^.

### 4.2. Collection of Fungal Endophytes and Fungal Growth Conditions

The root material of the *Oryza sativa* L. cultivar Puntal was obtained from paddy fields situated in the marshlands on the left bank of the Guadalquivir River. These fields are part of the Sartenejales (37°10′ N, 6°04′ W) and Casudis (37°08′ N, 6°05′ W) farms. The Sartenejales farm encompasses an area of 850 hectares, while the Casudis farm covers 2400 hectares. Both estates belong to the municipality of La Puebla del Río and are located at an elevation of merely 3 m above sea level, positioned to the south of Sevilla, Spain. The estuary of the Guadalquivir, in which the fields are situated, is influenced by tidal activity, resulting in the upstream penetration of seawater, which mixes with freshwater to create a transition zone characterized by variable salinity. Furthermore, irrigation water is frequently reused across numerous plots, contributing to increased water salinity [40]. During years of moderate irrigation, often due to water restrictions, the average salt concentration in irrigation water typically measures around 2.86 dS m^−1^. However, in years marked by severe restrictions, like in recent years, salinity levels can escalate to 4.0 dS m^−1^ or higher, leading to significant reductions in grain yield [41]. Puntal is the predominant rice variety cultivated in this region, classified as an *Indica*-type variety, and is grown in 60% of Andalusia. Although this cultivar is characterized as having medium sensitivity to salinity, it demonstrates a remarkable adaptability to the saline conditions prevalent in the Guadalquivir marshes. However, the potential role of endophytic microorganisms in facilitating this salt adaptation remains unclear.

To isolate endophytic fungi from the rice roots, segments of the root material were washed twice with distilled water and subsequently subjected to surface sterilization using 70% (*v*/*v*) ethanol for 2 min, followed by incubation in 2.5% (*w*/*v*) NaOCl and 0.1% (*v*/*v*) Triton-X100 for 5 min. The roots were then rinsed four times with sterile water. Next, the root fragments were directly plated on both water agar and 0.1 × Potato Dextrose Agar (PDA) containing 100 μg mL^−1^ ampicillin, 10 μg mL^−1^ tetracycline, and 150 μg mL^−1^ chloramphenicol, following previously established protocols [42,43,44]. To obtain pure isolates of endophytic fungi, they were transferred four times to fresh PDA plates supplemented with 150 μg mL^−1^ chloramphenicol.

In addition to the fungal strains isolated in this study, the previously described fungus *Fusarium* sp. strain K-23 was used [26]. The fungi were grown in darkness at a constant temperature of 28 °C on solidified PDA medium. Each week, the fungal cultures were refreshed.

### 4.3. Phylogenetic Classification of the Isolates

To achieve phylogenetic classification of the obtained fungal isolates, genomic DNA (gDNA) was extracted from fungal mycelia by applying the cetyltrimethylammonium bromide protocol [45]. The gDNA was subsequently used as the matrix to amplify the internal transcribed spacer (ITS) regions of the fungi by polymerase chain reaction (PCR), employing the primers ITS1 forward and ITS4 reverse [46]. The obtained DNA fragments were purified using the NucleoSpin Gel and PCR Clean-up Kit (Macherey-Nagel, Düren, Germany) according to the manufacturer’s protocol and subsequently Sanger sequenced by the StabVida Genetics Service (Caparica, Portugal) with their ITS1 and ITS4 primers. The taxonomic classification of the obtained amplicon sequences was performed using the UNITE Fungal ITS database v10.0 [28], displaying classifications at all taxonomic levels. This database encompasses identified fungal sequences with assignments to Species Hypothesis (SH) groups, which are defined based on variable sequence similarity thresholds [47]. Taxonomic assignments were subsequently verified and refined using the ITS region database of the NCBI (https://blast.ncbi.nlm.nih.gov/, accessed on 15 April 2026). Identity was assigned based on maximum homology and percent similarity, adhering to previously established criteria [48]. A phylogenetic tree was constructed using the neighbor-joining method [49] using the MEGA X v12.1 program [50]. The evolutionary distances were calculated with the maximum composite likelihood method [51]. The analysis incorporated 75 ITS sequences, comprising 5 sequences obtained in this study and 70 sequences sourced from the NCBI GenBank database (PRJNA177353) belonging to identified fungal orders. All positions containing gaps and missing data were eliminated. Evolutionary analyses were performed with 10,000 bootstrap replications.

### 4.4. Plant–Fungus Interaction and Plant Growth Promotion Assays

First, the capacity of the fungus to colonize *A. thaliana* roots was examined. For this, root tissue from inoculated and control plants was cut into 1 cm long segments, which were surface sterilized with 70% (*v*/*v*) ethanol for 2 min, followed by a washing step with 5% (*v*/*v*) NaOCl for 5 min, and three rinses with sterile distilled water. The explants were then placed in Petri dishes with solidified PDA medium and incubated for 5–7 days. Hyphal growth at the end of the root fragments was monitored.

To investigate the potential plant growth-promoting effects of the selected fungal isolates on Arabidopsis seedlings under both control and salt stress conditions, surface-sterilized seeds were plated and stratified as previously described. The plates were transferred to a growth chamber, where the seedlings were cultivated vertically for a period of 4 days. Subsequently, 5 seedlings were transferred to fresh square Petri dishes containing solidified Plant Nutrition Medium (PNM) [52], which either lacked NaCl (control) or contained either 50 mM or 75 mM NaCl (salt). The roots of each seedling were inoculated with 15 μL of a solution containing 1 × 10^5^ spores mL^−1^. Alternatively, each seedling was associated with a 5 mm medium plug extracted from either sterile PDA plates (mock) or from PDA plates harboring 1-week-old fungal mycelia (co-cultivation). The PNM plates were subsequently transferred to a growth chamber and maintained at 22–24 °C, with a 16/8 h photoperiod and a light intensity of 100 μmol photons m^−2^ s^−1^ for an additional 10 days. Finally, the plants were photographed for further phenotypic analysis before being dissected into root and shoot tissue to determine the fresh and dry weight of the plants.

### 4.5. RNA Isolation and Transcriptomics Analysis

For each growth condition, 100 mg of root tissue of 14 day-old mock- and fungus-inoculated seedlings (10 days post infection) that were grown in the absence and presence of 75 mM NaCl_2_, respectively, was harvested for total RNA extraction following the protocol of Oñate-Sánchez and Vicente-Carbajosa [53]. The total RNA was quantified utilizing a Nanodrop ND-1000 UV/V is spectrophotometer (Thermo Fisher Scientific, Waltham, MA, USA). RNA integrity was further assessed employing a Bioanalyzer 2100 (Agilent Technologies, Santa Clara, CA, USA) by the BMKGENE Genomics Service (Münster, Germany). The subsequent steps, including mRNA purification, library construction, and sequencing with 150-nucleotide paired-end reads on the NovaSeq™ 6000 (Illumina, San Diego, CA, USA) platform, were executed by the BMKGENE Genomics Service. They also conducted preliminary data analysis using their RNA sequencing (RNA-Seq) pipeline, which encompassed data filtering, quality control, and sequence alignment via HISAT2 v2.0.5 [54]. Transcript quantification was performed against the *A. thaliana* reference annotation (TAIR10, Ensembl release 45) using StringTie v3.0.0 [55]. For further RNA-Seq data analysis, the R-Shiny applications DIANE v1.0.6 [56] and iDEP v2.4.3 [57] were utilized. Differentially expressed genes (DEGs) were identified using the DESeq2 v1.20.0 algorithm [58], with a false discovery rate (FDR) threshold set to <0.05 and a log_2_(fold change) cut-off value of ≥1 and ≤−1, respectively (Supplementary Table S1). Venn diagrams were created using the Venn (http://bioinformatics.psb.ugent.be/webtools/Venn/, accessed on 25 April 2026) online tools to depict the overlaps and distinctions among DEGs across different datasets. Gene Ontology (GO) analyses were conducted using the ShinyGO (v0.85.1) [59] online tool. Heatmaps were generated with the ClustVis tool [60]. Each tissue, treatment, and condition was analyzed in triplicate biological samples.

### 4.6. Microscopic Inspection of Roots and Root Hairs

For the inspection of the root system architecture, the root system of at least 15 control and fungus-inoculated Arabidopsis seedlings at 10 days post infection was captured with a digital camera at a fixed distance of 29 cm. To assess root hair phenotypes, the different mock and fungus-treated Arabidopsis plants of at least 10 individual plants 7 days post infection were monitored. Images were captured using a Leica MZ10F stereo microscope equipped with a DFC 400C CCD camera. The length of the primary roots and the number of lateral roots were determined using ImageJ2 v2.14.0/1.54f [61].

### 4.7. Statistical Analysis

For the statistical assessment of data and the generation of plots, JASP v.0.96 (https://jasp-stats.org/) was utilized. A two-way ANOVA followed by a Tukey–Kramer post hoc test or Student’s *t*-test was conducted to perform statistical analysis of the data. Sample sizes (n) for each experiment are provided in the respective figure legends. Results were considered statistically significant when the *p*-value was less than or equal to a value of 0.05.

## Data Availability

All data supporting the findings of this study are available within the paper and its supplementary data. The full RNA-Seq dataset for the mock and *Fusarium* sp. strain K-23 inoculated plants in the presence and absence of salt is available at the GEO functional genomics data repository, dataset GSE260960.

## Supplementary Materials

The following supporting information can be downloaded at: https://www.mdpi.com/article/10.3390/ijms27177590/s1.
