# Supplementary material for: Habitat-Adapted Fungal Symbionts Promote Salt Stress Tolerance Through Distinct Root Mechanisms and Shared Shoot Regulatory Networks in Arabidopsis thaliana
Source: Int J Mol Sci. 2026 Aug 25;27(17):7590. doi: 10.3390/ijms27177590 (PMC13565824; doi:10.3390/ijms27177590)
Supplement: Supplementary file 1 [file ijms-27-07590-s001.zip › Supplementary Figures.pdf]

**Supplementary Figure S1:**

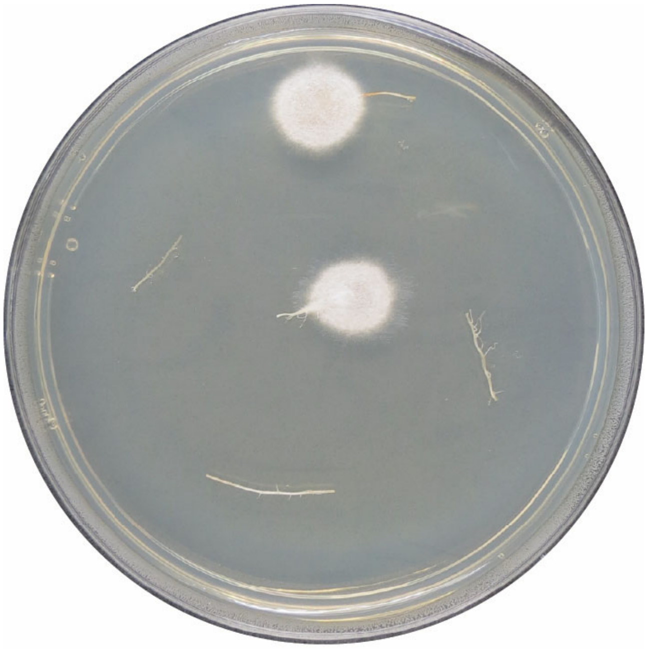


**Supplementary Figure S1.** Re-isolation of *Reticulascus* sp. strain S5 from Arabidopsis roots. Arabidopsis roots inoculated with *Reticulascus* sp. strain S5 were harvested at 7 dpi and subjected to thorough surface sterilization. Subsequently, root fragments measuring 1 cm in length were placed on PDA media and incubated for several days at 28°C in darkness. The emergence of fungal hyphae from the ends of the root fragments indicates successful colonization root of the root endosphere.

**Supplementary Figure S2:**

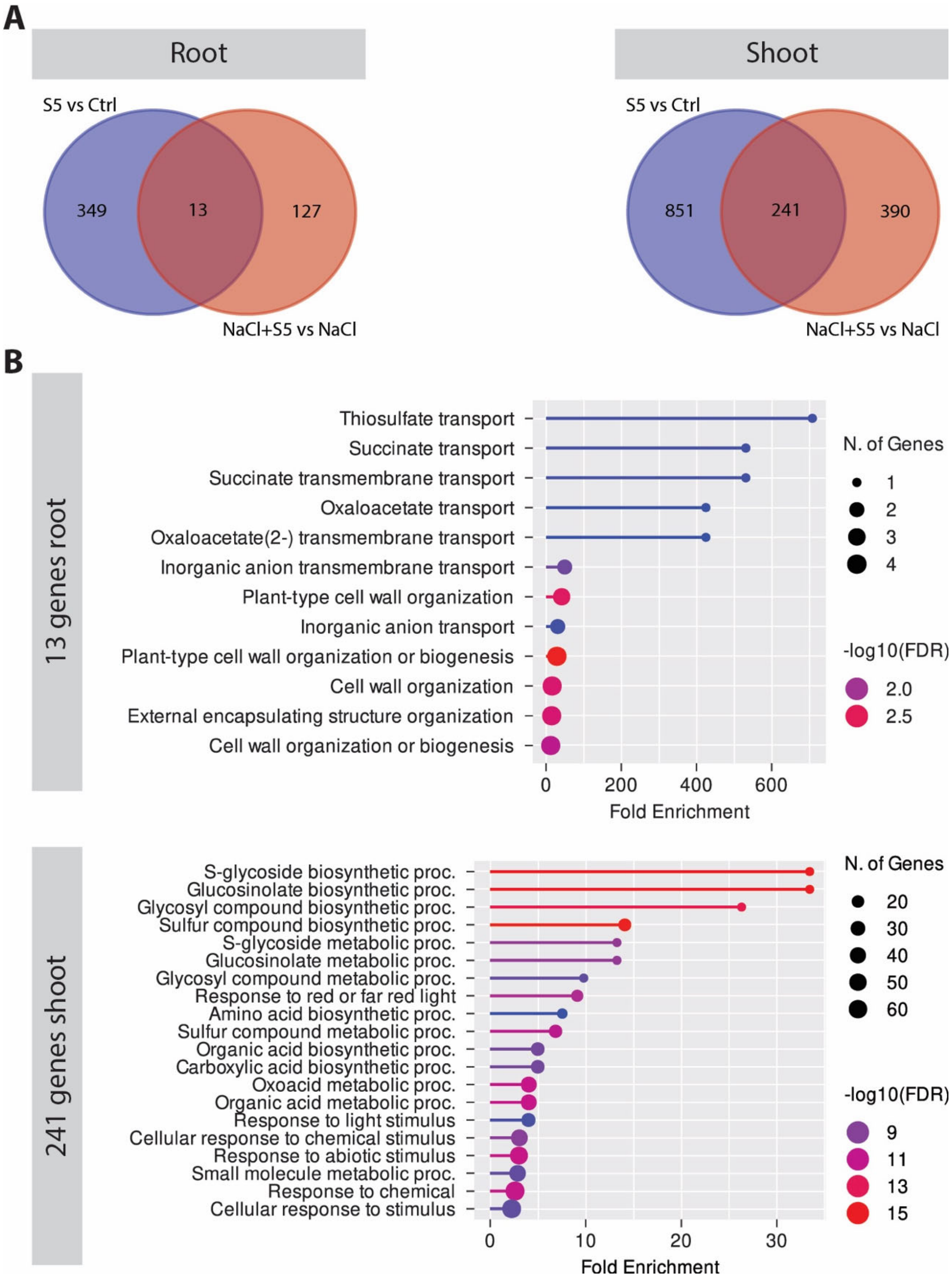


**Supplementary Figure S2.** Comparison of DEGs only affected by *Reticulascus* sp. strain S5 inoculation in presence and absence of 75 mM NaCl for root and shoot tissues. (**A**) Venn diagram analysis comparing DEGs of the S5 versus Ctrl condition with those of the NaCl+S5 versus NaCl condition. (**B**) GO enrichment

analysis of the 13 shared DEGs in roots and the 241 shared DEGs in shoots of Arabidopsis plants grown either in the presence or absence of salt stress. Each circle in the figure represents a distinct GO term. The circle size indicates the number of genes enriched in the corresponding GO term. The significance of the observed gene enrichment is represented by a colour gradient referring to the -log10(FDR) value.
